# Association of chronic musculoskeletal pain, APOE Ɛ4 genotype, and analgesics with the risk of dementia: a population-based prospective cohort study

**DOI:** 10.1097/JS9.0000000000002348

**Published:** 2025-03-18

**Authors:** Yuan Zhang, Jiangtao Feng, Hongxi Yang, Shu Li

**Affiliations:** a Department of Pathology, Raymond G. Perelman Center for Cellular and Molecular Therapeutics, Children’s Hospital of Philadelphia, Philadelphia, PA, USA; b Department of orthopedic, Tianjin NanKai Hospital, Tianjin, China; c Department of Bioinformatics, School of Basic Medical Sciences, Tianjin Medical University, Tianjin, China; d School of Management, Tianjin University of Traditional Chinese Medicine, Tianjin, China

## Abstract

Chronic musculoskeletal pain (CMP) is associated with the risk of dementia, yet little is known about whether this association is modified by APOE genotype and whether analgesics may mitigate the risk effect of CMP on dementia. This prospective cohort study, included 415 072 participants from UK Biobank, found that CMP was associated with a higher risk of dementia, and this association may be modified by the APOE genotype. Analgesics may not mitigate the risk effect of CMP on dementia.

**HIGHLIGHTS**

- **What is already known on this topic?**Previous research has established a link between chronic musculoskeletal pain (CMP) and dementia, but the interaction with the APOE ε4 genotype and the role of analgesics in mitigating this risk remain unclear, necessitating further investigation.
- **What this study adds?**This study provides novel evidence indicating that the APOE ε4 genotype may modify the association between CMP and dementia. Additionally, it suggests that analgesics, particularly ibuprofen and paracetamol, may not effectively mitigate the risk of dementia associated with CMP.
- **How this study might affect research, practice, or policy?**These findings underscore the importance of considering genetic factors, such as the APOE ε4 genotype, in understanding the relationship between CMP and dementia. Furthermore, we raise questions about the efficacy of analgesics in managing dementia risk associated with CMP, which may influence clinical practice and healthcare policy in pain management and dementia prevention.

*Dear Editor*,

Dementia is the seventh leading cause of death globally^[^1^]^. With the aging population, the number of people with dementia is projected to reach 152 million by 2050^[^1^]^. Despite advances in molecular neuroimaging, understanding of clinicopathologic correlations, and the development of novel biomarkers, clinical treatment of dementia remains suboptimal. Identifying individuals at high risk of dementia may contribute to its prevention and early treatment.

Chronic musculoskeletal pain (CMP) is prevalent among older adults and contributes to functional decline and muscle weakness^[^2^]^. A systematic review reported a prevalence of CMP of 26% in the general population and 39% among individuals aged >65 years^[^3^]^. Pain, loss of function, and disability due to CMP impose substantial physical, psychosocial, and socioeconomic burdens on patients and their families^[^3^]^. Population-based studies suggested an association between CMP and dementia^[^4^]^. CMP induces systemic inflammation, oxidative stress, and behavioral changes (e.g., physically inactive, depression), which could interact with APOE ɛ4-related pathways to accelerate cognitive decline^[^5,6^]^. The APOE genotype is linked to neuroinflammation and subsequent amyloid-beta aggregation, which may exacerbate neurodegeneration in the context of chronic pain. Moreover, prior evidence suggests APOE polymorphisms are associated with pain sensitivity^[^7^]^, further supporting shared mechanisms. However, whether the association between CMP and dementia is modified by the APOE genotype, the strongest genetic risk factor for dementia, remains unclear.

Pain research has proposed pain relief as the termination of negative affect and return to a neutral state^[^8^]^. Analgesics, the primary treatment for CMP, can ease mental tension caused by pain, divert attention away from pain, and reduce rumination. We hypothesize that analgesics may mitigate the risk effect of CMP on dementia. Therefore, the purpose of this study was to examine whether the association between CMP and dementia is modified by the APOE genotype and to explore whether analgesics may mitigate the effect of CMP on dementia risk.

The UK Biobank is a population-based cohort study that recruited more than 500 000 participants (aged 37–73 years) who attended one of 22 assessment centers across the UK between 2006 and 2010 and were followed up to 2021^[^9^]^. Of the 502,412 participants, 87,340 were excluded, including 228 with prevalent dementia, 16 740 with missing genetic and/or CMP data, 69 122 with chronic pain in other locations (e.g., headache, facial pain, or stomach) at baseline, and 1250 lost to follow-up. Finally, 415 072 participants were included in the analysis.

Outcomes, including incident dementia, Alzheimer’s disease (AD), and vascular dementia (VAD), were ascertained through self-reports, hospital admissions, and death records. Participants reporting pain persisting for ≥3 months at any site were classified as having CMP, defined as pain in the neck, shoulder, back, hip, or knee. The number of CMP locations was categorized as 0–4. The APOE genotype, a key genetic factor in dementia risk, was coded as APOE ɛ4 non-carriers or APOE ɛ4 carriers.

Cox proportional hazards regression models were applied to estimate the hazard ratios (HRs) and 95% confidence intervals (CIs) of dementia risk in relation to CMP, APOE ε4 genotype, and analgesic use. Multiplicative interaction between CMP and the APOE ɛ4 genotype was assessed using a cross-product term in the model to determine whether the absolute risk attributable to CMP differs between APOE ɛ4 carriers and non-carriers. Additive interaction was evaluated by estimating the relative excess risk due to interaction (RERI), the attributable proportion (AP), and the synergy index (SI), indicating whether the combined effect of CMP and APOE ɛ4 on dementia risk is greater than expected based on their individual effects. All models were adjusted for sex, age, ethnicity, education level, socioeconomic status, employment status, alcohol consumption, physical activity, smoking status, diet, BMI, heart disease, stroke, hypertension, diabetes, depression, cholesterol levels, C-reactive protein levels, analgesics, and the APOE ɛ4 genotype. All analyses were performed using R. Statistical significance was defined as a two-sided *P*-values < 0.05.

Over a median of 12.7 (inter quartile range [IQR]: 12.1–13.4) years, 6,239 individuals developed dementia (2656 and 1363 cases of AD and VAD, respectively). In multi-adjusted Cox regression models, a dose–response relationship was observed between the number of CMP location and dementia risk (*P* for trend < 0.001, Table 1). Each additional CMP location increased the risk of incident dementia (HR, 1.10; 95% CI: 1.08–1.13). In multi-adjusted competing risk regression models, compared to individuals without CMP, the HRs (95% CIs) among those with 1, 2, 3, and 4 CMP locations were 1.09 (1.03–1.16), 1.21 (1.12–1.31), 1.38 (1.23–1.54), and 1.52 (1.27–1.81) for dementia risk, respectively.

**Table 1:** Association between CMP locations and the risk of incident dementia: results from Cox regression and competing risk regression models.

	Incident dementia			Incident Alzheimer’s disease			Incident vascular dementia	
	HR (95% CI)^a^	*P-*value		HR (95% CI)^a^	*P* value		HR (95% CI)^a^	*P-*value
Cox regression models								
CMP locations								
Neck/shoulder	1.20 (1.12–1.29)	<0.001		1.25 (1.13–1.39)	<0.001		1.02 (0.88–1.19)	0.768
Back	1.27 (1.19–1.36)	<0.001		1.24 (1.12–1.37)	<0.001		1.15 (1.00–1.32)	0.056
Hip	1.24 (1.14–1.34)	<0.001		1.24 (1.09–1.41)	<0.001		1.08 (0.91–1.29)	0.249
Knee	1.17 (1.10–1.25)	<0.001		1.18 (1.07–1.31)	0.001		1.09 (0.94–1.25)	0.370
CMP score, categorical								
0	1.00 (ref.)			1.00 (ref.)			1.00 (ref.)	
1	1.08 (1.02–1.15)	0.010		1.09 (0.99–1.20)	0.078		1.04 (0.92–1.19)	0.530
2	1.20 (1.10–1.30)	<0.001		1.22 (1.08–1.39)	0.001		0.99 (0.83–1.18)	0.876
3	1.37 (1.23–1.53)	<0.001		1.29 (1.08–1.54)	0.005		1.11 (0.87–1.41)	0.411
4	1.52 (1.27–1.81)	<0.001		1.67 (1.27–2.19)	<0.001		1.36 (0.95–1.95)	0.096
Competing risk regression models								
CMP locations								
Neck/shoulder	1.20 (1.12–1.28)	<0.001		1.23 (1.11–1.37)	<0.001		1.02 (0.88–1.19)	0.760
Back	1.29 (1.21–1.37)	<0.001		1.24 (1.12–1.37)	<0.001		1.17 (1.02–1.34)	0.030
Hip	1.25 (1.15–1.36)	<0.001		1.23 (1.09–1.40)	0.001		1.11 (0.93–1.31)	0.250
Knee	1.19 (1.11–1.27)	<0.001		1.19 (1.07–1.31)	0.001		1.10 (0.96–1.27)	0.170
CMP score, categorical								
0	1.00 (ref.)			1.00 (ref.)			1.00 (ref.)	
1	1.09 (1.03–1.16)	0.004		1.09 (0.99–1.20)	0.070		1.06 (0.93–1.21)	0.370
2	1.21 (1.12–1.31)	<0.001		1.22 (1.08–1.38)	0.001		1.01 (0.85–1.21)	0.890
3	1.38 (1.23–1.54)	<0.001		1.28 (1.07–1.53)	0.007		1.13 (0.89–1.44)	0.320
4	1.52 (1.27–1.81)	<0.001		1.64 (1.25–2.16)	<0.001		1.39 (0.97–2.01)	0.073

^a^ Cox regression and competing risk regression models were adjusted for sex, age, ethnicity, education level, socioeconomic status, employment status, alcohol consumption, smoking status, physical activity, diet, BMI, heart disease, stroke, hypertension, diabetes, depression, cholesterol, analgesics, and APOE genotype.

There was a statistically significant multiplicative interaction between CMP and the APOE genotype on the incidence of dementia (*P* = 0.006). Each additional CMP location was associated with a higher dementia risk in APOE ɛ4 carriers (HR, 1.13, 95% CI: 1.09–1.17) compared to non-carriers (HR, 1.07, 95% CI: 1.03–1.11; Figure 1). While the absence of a significant additive interaction (RERI: 0.634, 95% CI: −0.041 to 1.049; AP: 0.212, 95% CI: −0.138 to 0.308; SI: 1.257, 95% CI: −0.227 to 1.755) suggests that CMP and APOE ɛ4 do not act synergistically on an absolute risk scale. This indicates that while the relative risk of dementia differs between genetic subgroups, the combined effect does not exceed the sum of individual risks in absolute terms.

**Figure 1.**
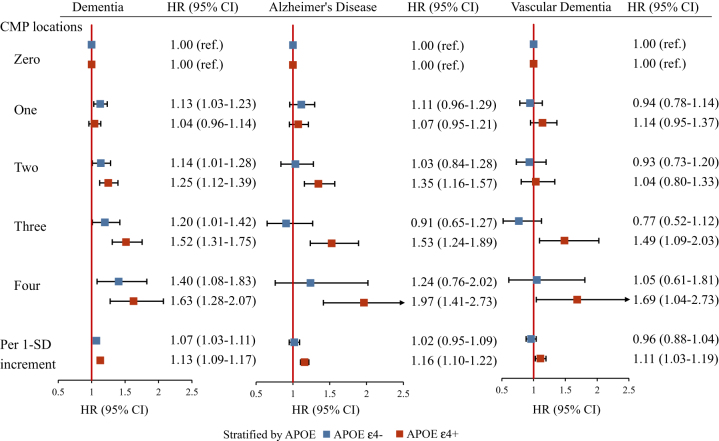
Multi-adjusted hazards ratios (HRs) and 95% confidence intervals (CIs) for dementia according to the number of chronic musculoskeletal pain (CMP) locations, stratified by the presence of the APOE ɛ4 genotype. Note: Cox regression models were adjusted for sex, age, ethnicity, education level, socioeconomic status, employment status, alcohol consumption, smoking status, physical activity, diet, body mass index (BMI), heart disease, stroke, hypertension, diabetes, depression, cholesterol, and analgesic use.

We further examined whether analgesics may mitigate the risk effect of CMP on dementia. Compared to participants without CMP, the HR (95% CIs) for dementia was 1.15 (1.09–1.23) for those with CMP not using analgesics, 1.14 (0.99–1.31) for those with CMP and using ibuprofen alone, 1.34 (1.22–1.46) for those with CMP and using paracetamol alone, and 1.49 (1.28–1.73) for those with CMP and using both ibuprofen and paracetamol (Fig. 2).

**Figure 2.**
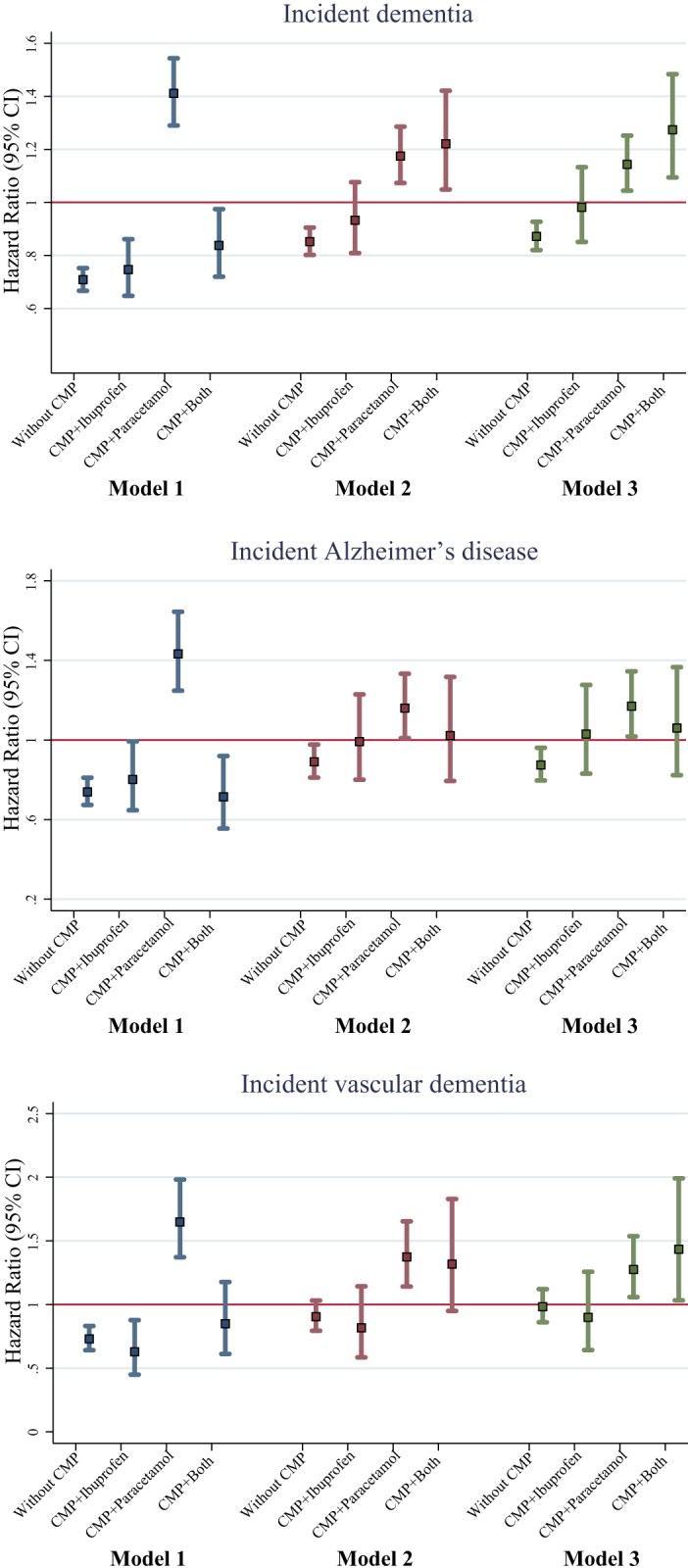
Multi-adjusted HRs and 95% CIs for dementia according to joint exposure to CMP and analgesics. Note: Cox regression models were adjusted for sex, age, ethnicity, education level, socioeconomic status, employment status, alcohol consumption, smoking status, physical activity, diet, BMI, heart disease, stroke, hypertension, diabetes, depression, cholesterol, and APOE ɛ4 genotype.

This large population-based cohort study found that the association between CMP and dementia may be modified by APOE ɛ4 genotype. Additionally, analgesics (ibuprofen and paracetamol) may not mitigate CMP-related dementia risk. These findings highlight the need for early cognitive screening in individuals with CMP, particularly those with multiple pain sites or the APOE ɛ4 genotype. Personalized medicine strategies could leverage genetic and clinical risk profiling to identify high-risk individuals and tailor prevention efforts accordingly. While further research is needed to evaluate its feasibility, cost-effectiveness, and clinical impact.

The mechanisms underlying the multiplicative interaction between the APOE ε4 genotype in dementia and CMP are multifactorial and incompletely understood. Neurophysiological and neuroimaging studies confirmed that older adults are particularly susceptible to the negative effects of pain. CMP may induce behavioral and psychological changes, such as smoking, physical inactivity, anxiety, and depression, which interact with dementia-related genetic factors^[^6,10^]^. Polymorphisms in the APOE gene are also associated with CMP^[^7^]^, suggesting a shared role in dementia pathogenesis.

Pain research has proposed pain relief as the termination of negative affect and the return to a neutral state. While ibuprofen and paracetamol are effective for musculoskeletal pain^[^11^]^, our findings suggest they may not mitigate CMP-related dementia risk. CMP management is challenging, especially in older adults with comorbidities. While analgesics effectively relieve pain, they may not address CMP-related dementia risk. This underscores the importance of exploring alternative or complementary pain management strategies, such as non-pharmacological interventions, to address both pain and cognitive health. Previous studies have reported that non-pharmacological interventions, such as physical therapy, cognitive-behavioral therapy, and exercise programs, have shown promise in both pain relief and cognitive^[^12-14^]^. These strategies may provide a dual benefit, addressing both the burden of CMP and potential neurocognitive decline.

Although the link between CMP and dementia is well-documented, our study is among the first to investigate whether this relationship is modified by APOE ɛ4 genotype. Moreover, our findings indicate that analgesic use does not significantly attenuate the association between CMP and dementia. In fact, certain analgesic use patterns, particularly paracetamol alone or in combination with ibuprofen, were associated with a higher dementia risk. This suggests that analgesic use may reflect more severe pain rather than providing neuroprotective effects, an important clinical consideration for pain management in older adults. Additionally, our results highlight the need for alternative pain management strategies beyond conventional analgesics, such as non-pharmacological interventions, to address both pain and cognitive health. This study also had several limitations. Early cognitive decline may contribute to increased pain perception and analgesic use, rather than CMP leading to dementia, which could result in reverse causation. Additionally, although all analyses in the present study were adjusted for known potential sources of bias, the possibility of unmeasured confounding factors remains. Additionally, analgesic use may reflect more severe CMP, and the lack of adjustment for CMP severity may confound the observed association between analgesic use and dementia risk. However, our study limited by not accounting for the severity of CMP. Future studies should prioritize integrating validated pain severity metrics or using alternative proxies such as prescription patterns and clinical pain assessments to disentangle the effects of analgesics from underlying pain intensity.

**Supplementary materials**

Supplementary Methods, Tables S1–S10, Figure S1. http://links.lww.com/JS9/E23

## Data Availability

The data that support the findings of this study are available from UK Biobank (https://www.ukbiobank.ac.uk/), but restrictions apply to the availability of these data, which were used under license for the current study, and so are not publicly available. Data are however available from the authors upon reasonable request and with permission of UK Biobank.
